# Pathophysiology of Skin Resident Memory T Cells

**DOI:** 10.3389/fimmu.2020.618897

**Published:** 2021-02-03

**Authors:** Yoshiki Tokura, Pawit Phadungsaksawasdi, Kazuo Kurihara, Toshiharu Fujiyama, Tetsuya Honda

**Affiliations:** ^1^ Department of Dermatology, Hamamatsu University School of Medicine, Hamamatsu, Japan; ^2^ Department of Cellular & Molecular Anatomy, Hamamatsu University School of Medicine, Hamamatsu, Japan

**Keywords:** skin, resident memory T cell, skin immunity, psoriasis, vitiligo, cutaneous T cell lymphoma, fixed drug eruption

## Abstract

Tissue resident memory T (T_RM_) cells reside in peripheral, non-lymphoid tissues such as the skin, where they act as alarm-sensor cells or cytotoxic cells. Physiologically, skin T_RM_ cells persist for a long term and can be reactivated upon reinfection with the same antigen, thus serving as peripheral sentinels in the immune surveillance network. CD8^+^CD69^+^CD103^+^ T_RM_ cells are the well-characterized subtype that develops in the epidermis. The local mediators such as interleukin (IL)-15 and transforming growth factor (TGF)-β are required for the formation of long-lived T_RM_ cell population in skin. Skin T_RM_ cells engage virus-infected cells, proliferate *in situ* in response to local antigens and do not migrate out of the epidermis. Secondary T_RM_ cell populations are derived from pre-existing T_RM_ cells and newly recruited T_RM_ precursors from the circulation. In addition to microbial pathogens, topical application of chemical allergen to skin causes delayed-type hypersensitivity and amplifies the number of antigen-specific CD8^+^ T_RM_ cells at challenged site. Skin T_RM_ cells are also involved in the pathological conditions, including vitiligo, psoriasis, fixed drug eruption and cutaneous T-cell lymphoma (CTCL). The functions of these T_RM_ cells seem to be different, depending on each pathology. Psoriasis plaques are seen in a recurrent manner especially at the originally affected sites. Upon stimulation of the skin of psoriasis patients, the CD8^+^CD103^+^CD49a^-^ T_RM_ cells in the epidermis seem to be reactivated and initiate IL-17A production. Meanwhile, autoreactive CD8^+^CD103^+^CD49a^+^ T_RM_ cells secreting interferon-γ are present in lesional vitiligo skin. Fixed drug eruption is another disease where skin T_RM_ cells evoke its characteristic clinical appearance upon administration of a causative drug. Intraepidermal CD8^+^ T_RM_ cells with an effector-memory phenotype resident in the skin lesions of fixed drug eruption play a major contributing role in the development of localized tissue damage. CTCL develops primarily in the skin by a clonal expansion of a transformed T_RM_ cells. CD8^+^ CTCL with the pagetoid epidermotropic histology is considered to originate from epidermal CD8^+^ T_RM_ cells. This review will discuss the current understanding of skin T_RM_ biology and their contribution to skin homeostasis and diseases.

## Introduction

The number of T cells infiltrating in the skin is nearly twice as many as that in the peripheral blood, and the majority of these cells are effector memory T cells (1). T cells in the skin include αβ T cells accounting for up to 99% and γδ T cells for around 1% (2). Thus, the skin is a homing organ for T cells in physiological and pathological conditions related to adaptive immune response. Before the discovery of resident memory T (T_RM_) cells, it was supposed that T cells infiltrating in inflamed or infected tissue transiently reside and undergo apoptosis or exit the tissue after clearance of inflammation or infection. Skin T_RM_ cells are a memory T cell subset that provides local surveillance and do not migrate out of the skin. This memory subset has distinct behavior and transcriptional profile that distinguish T_RM_ cells from other memory T cell compartment.

Tissue T_RM_ cells reside in peripheral, non-lymphoid tissues such as the skin, where they act as alarm-sensor cells or cytotoxic cells (3, 4). Physiologically, skin T_RM_ cells persist for a long term and can be reactivated upon reinfection with the same antigen, thus serving as a part of an immune surveillance network. CD8^+^CD69^+^CD103^+^ T_RM_ cells are the well-characterized subtype that develops in the epidermis, although CD4^+^ T_RM_ cells are documented in certain conditions. Local signaling by IL-15 and TGF-β is required for the formation of these long-lived memory cells (5).

Skin T_RM_ cells play a critical defensive role against skin infections. In addition to this essential physiological role, they are also involved in the pathological conditions (6), as exemplified by psoriasis. The functions of these T_RM_ cells seem to be different, depending on each skin disease. The T_RM_ cell-inducing skin diseases have currently extended from fixed drug eruption to psoriasis and cutaneous T-cell lymphoma, and even to vitiligo. In this review, we will discuss recent insights into skin T_RM_ cells, with emphasis on their pathogenic roles in these heterogeneous skin disorders.

## Tissue T_RM_ Cells

T_RM_ cells, which lack the ability of recirculation *via* the bloodstream and reside in the tissue, exist in various tissues in various organs. However, the phenotypes of T_RM_ cells in each tissue, such as surface markers, the longevity, and the signals for their survival are not uniform and highly heterogeneous. Insights into T_RM_ cells in various tissues have mostly been obtained from mouse studies, and the data of human T_RM_ cells are relatively scarce, because of the technical difficulties in obtaining samples and taking enough number of cells from small biopsy samples in human. It is considered that both CD8^+^ T_RM_ and CD4^+^ T_RM_ cells exist, but the property is best defined for CD8^+^ T_RM_ cells. In this section, we will briefly introduce the characteristics of T_RM_ cells in various tissues, mainly focusing on CD8^+^ T_RM_ cells in mice (**Table 1**).

**Table 1:** Resident memory T cells in various tissues in mice and humans.

Tissue of residency	Type of T_RM_ reported in mice or human		Possible involvements in human diseases
	CD4 T_RM_	CD8 T_RM_	
Skin		✓	Fixed drug eruption (7)
		✓	Psoriasis (8)
		✓	Vitiligo (9)
		✓	Alopecia areata (10)
		✓	HSV infection (11)
	✓		Candida infection (12)
	✓		Leishmania infection (13)
	✓	✓	CTCL (14)
Gut	✓	✓	Inflammatory bowel disease (15, 16)
Lung	✓	✓	Influenza (17)
	✓	✓	RSV infection (18)
	✓		Allergic asthma (19)
Synovial bursa	✓	✓	Rheumatoid arthritis (20)
Central nervous system		✓	Multiple sclerosis (21)
		✓	Schizophrenia (22)
Kidney		✓	Lupus nephritis (23, 24)

The surface markers and longevity of CD8^+^ T_RM_ cells are critical issues and have been studied in mouse tissues. One of the most important functions of T_RM_ cells is the defense against pathogens such as viruses, bacteria, fungi, and parasites, all of which commonly invade to our body through barrier tissues. Consistently, T_RM_ cells are observed in barrier tissues such as the skin, intestines, lung, and female reproductive tract (25, 26). T_RM_ cells are also detected in non-barrier tissues such as the central nervous system, liver, and salivary glands (25, 26). Furthermore, T_RM_ cells are present in lymphoid tissues, some of which are derived from non-lymphoid tissues (27). CD69 and CD103 are the key surface markers of T_RM_ cells in general, however, the expression patterns of these markers are various depending on the tissues, and even show heterogeneity in the same tissue. CD103 is expressed in T_RM_ cells in most tissues such as the skin and central nervous system, but T_RM_ cells lacking CD103 have been reported in some tissues including intestines (28) and liver (29). CD69, a C-type lectin, is expressed in most T_RM_ cells. CD69 is supposed to work as a stop signal that prevents tissue egress of T_RM_ cells by antagonizing sphingosine-1-phosphate receptor 1 (S1PR1). However, a substantial proportion of T_RM_ cells in the pancreas, salivary glands, and female reproductive tract was reported to be negative for both CD69 and CD103 (30).

Longevity, which can be defined as the persistence of T_RM_ cells in the tissues, may be also quite different between tissues (4). It has been reported that T_RM_ cells in the lungs and liver persist for weeks to months (31, 32), while T_RM_ cells in the skin remain numerically stable for months to years (33–35), suggesting a tissue specificity of longevity. Longevity is the net effects of several factors such as recruitment, maintenance, division, death, egress, and competition. The extent of the effects of each factor is various depending on the tissues. For example, at the steady state, the ratio of T_RM_ cells that uptake BrdU over 7 days is 0%–5% in the lung (36) and skin (37), while Ki67^+^ T_RM_ cells in the brain is reported around 9% (38), suggesting the various proliferation ability of T_RM_ cells depending on the tissues. As for the maintenance signals of T_RM_ cells, IL-15 is one of the most important one. Indeed, IL-15 is required for the maintenance of T_RM_ cells in the skin (39), liver (40), salivary glands and kidney (41). However, this is not the case for T_RM_ cells in the female reproductive tract, pancreas, small intestines, and secondary lymphoid organs (SLOs) (41). Expression of CD103 may also be important for the persistence of T_RM_ cells in several tissues such as the skin (39) and the gut (42). TGF-β is necessary for the development of T_RM_ cells in the skin (39), gut (43), and lung (44), while not required for the development of T_RM_ cells in lamina propria of intestine (28). Thus, T_RM_ cells in each tissue possess their own characteristics. Because the environment in each tissue such as available cytokines and nutrients are various, T_RM_ cells seem to adapt to unique local environment to survive.

In human, T cells showing surface markers similar to murine T_RM_ cells have been detected in various tissues, suggesting that T_RM_ cells also exist in human. It is considered that T_RM_ cells play crucial roles for the protection of the host against pathogens, as well as the development of inflammatory diseases. T_RM_ cells in the skin are probably the best studied population in human T_RM_ cells. In the genital skin after human simplex virus (HSV) infection, virus-specific CD8^+^ T cells persist at the epidermal-dermal junction (11). Involvement of T_RM_ cells is suggested in the development of various inflammatory skin diseases, such as psoriasis, vitiligo, and drug eruption, which will be discussed later. T_RM_ cells are also detected in the gut, and are suspected to contribute to the development of Crohn’s disease (15). In the lung, CD69^+^ or CD103^+^ CD8^+^ T_RM_-like cells are detected in patients with influenza or respiratory syncytial virus infection (17, 18). Other than these tissues, existence of T_RM_ cells has been reported in the female reproductive tract after the vaccination targeting human papilloma virus 16 (45) and liver in hepatitis C infection (46), suggesting the importance of T_RM_ cells in the protective immunity in human as well.

CD4^+^ T_RM_ cells are usually found within the tissue parenchyma, such as the dermis in the skin. Compared with CD8^+^ T_RM_ cells, little is known about the characteristics and functions of CD4^+^ T_RM_ cells. However, this subset may also play important roles in the protective immunity against pathogens in several tissues (47). In mice, the protective roles of CD4^+^ T_RM_ cells have been reported in *Leishmania major* infection in the skin (48), herpes simplex virus infection in the genital mucosa (34), *Chlamydia trachomatis* infection at the reproductive mucosa (49), and *Streptococcus pneumonia* infection in the lung (50). It remains to be clarified whether those CD4^+^ T_RM_ cells are really resident in tissues or just a subset of memory CD4^+^ T cells which spend an extended period time in the tissue before circulation.

## Identification and Definition of Skin T_RM_ Cells

**Figure 1:**
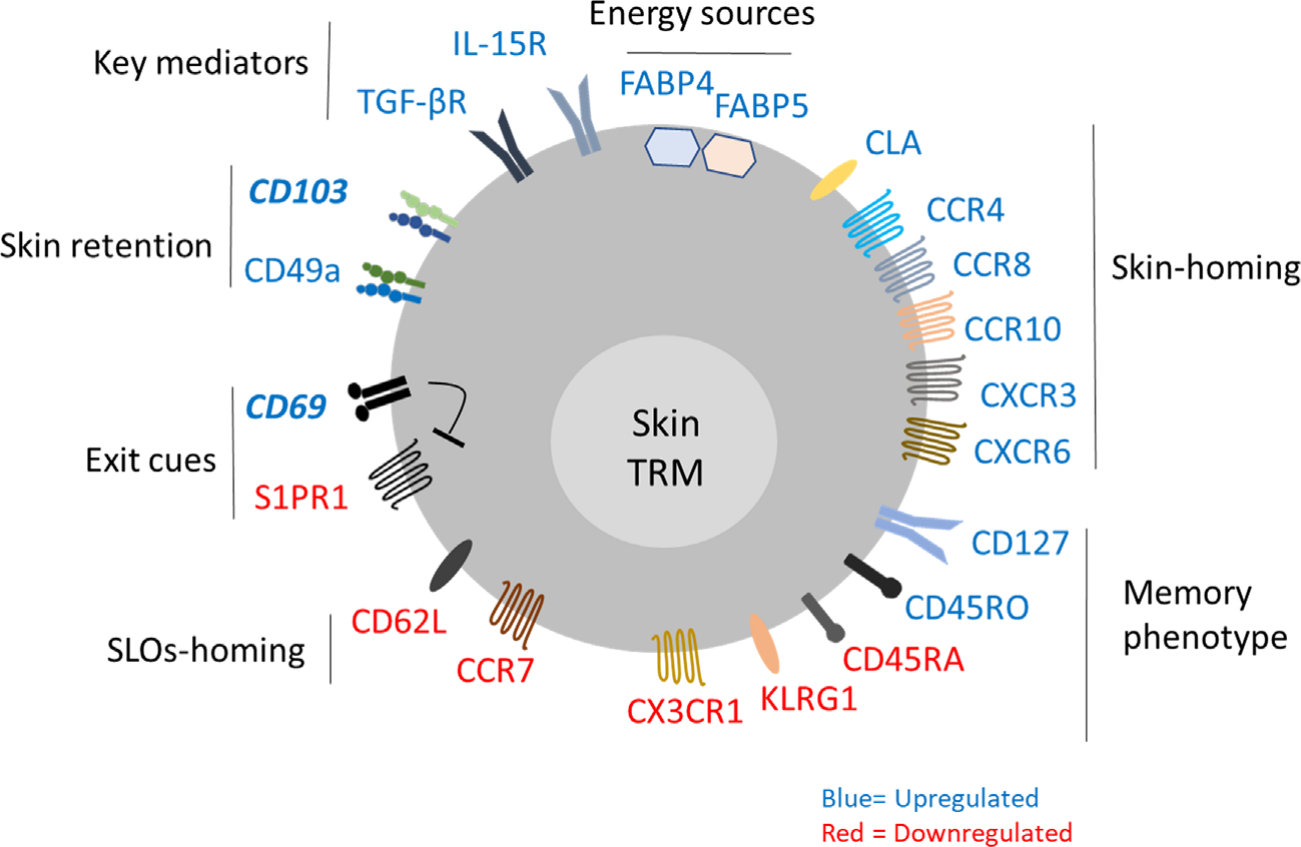
Characterization of skin resident memory T cell.

As discussed above, the markers that identify tissue T_RM_ cells may differ among the tissues. The characteristic behavior and markers of skin T_RM_ were well studied in murine models. In human, it is technically difficult to address the migratory behavior of skin T_RM_ cells in an *in vivo* system. The resident memory properties of human skin T cells are largely described on CD8^+^ T cells with surface markers similar to those of murine T_RM_ cells (23, 51). In this section, we review the current evidence of skin T_RM_ identification, which mostly came from the murine study, and their relevance in human **(** **Figure 1**).

### Precursors of Skin T_RM_ Cells

Naïve CD8^+^ T cells proliferate and differentiate into a pool of effector cells upon recognition of cognate antigen. During the effector phase, CD8^+^ effector cells can be divided into short-lived effector cells (SLECs) and memory precursor effector cells (MPECs) (52). SLECs are characterized by KLRG1^hi^ IL-7Rα^lo^(CD127), while MPECs are KLRG1^lo^ IL-7Rα^hi^. The fate decision of SLECs/MPECs depends on a sum of inflammatory signals that create a T-bet gradient, in which a low-level magnitude promotes MPECs fate during T cell priming (52). Almost all SLECs undergo apoptosis, whereas MPECs turn into heterogenous populations of long-lived memory CD8^+^ T cells after clearance of infection (52). In early skin infection of herpes simplex virus, skin-infiltrating T cells are mainly KLRG1^+^ effector cells, while at the memory phase, the remaining memory T cells in the skin bear negative or low expression of KLRG1. Consistently, the adoptive transfer study of KLRG1^-^ T cells confirmed that KLRG1^-^ MPECs gave rise to T_RM_ cell populations in the skin (39). Memory T cells also express CD45RO but not CD45RA. Skin-infiltrating T cells isolated from normal human skin were almost all CD45RO^+^ memory T cells (1). Collectively, skin T_RM_ cells possess the memory precursor phenotype, KLRG1^-^CD127^+^CD45RO^+^CD45RA^-^.

### Skin-Homing Molecules on T_RM_ Cells

Skin-infiltrating memory T cells express a distinct homing receptor called cutaneous lymphocyte-associated antigen (CLA), which binds to E-selectin and P-selectin and allowing CLA^+^ T cells to enter the skin (1). Nearly all CLA^+^ effector memory T cells are resident in human skin during steady state (1). Chemokine receptor (CCR)10 is one of the essential chemokine receptors for skin homing of T cells (53), as CCR10-deficient mice showed a reduction of CD8^+^ T cells in the skin (54). Similarly, CD8^+^ T cells lacking CCR10 impaired their T_RM_ forming capacity (55). CXCR6 is expressed on skin T_RM_ cells in human (1) and mice (56), and CXC chemokine ligand (CXCL)16, a ligand for CXCR6, is expressed on epidermal keratinocytes and can be released as a chemoattractant (57). T cells lacking CXCR6 had low capacity to form T_RM_ cells in the skin, whereas CXCR6^-/-^ and wild-type T cells were not different in number in the SLOs. Consistently, direct injection of CXCR6^-/-^ CD8^+^ T cells into the skin also decreased T_RM_ formation, suggesting that CXCR6 is important for retention rather than recruitment of CD8^+^ T cells to the skin (55). CCR4 is an essential skin-homing molecule for the migration of T cells to the skin (58) and highly expressed on skin T_RM_ cells (1). Mogamulizumab, a humanized anti-CCR4 antibody, was approved for mycosis fungoides (MF) and Sézary syndrome (SS), which are a malignancy of skin-homing malignant T cells (59). However, the exact role of CCR4 on skin CD8 T_RM_ formation is not clear. Previous studies showed that CXCR3 expression is necessary for T_RM_ cell precursors to enter the epidermis, and CD8^+^ T cells lacking CXCR3 resulted in less formation of CD103^+^ T_RM_ cells in mice (39). Skin CCR8^+^ T cells show phenotypic, functional, and transcriptomic profiles compatible with T_RM_ cells (60). CCR8 is expressed on half of cutaneous memory T cells, whereas very few CCR8 is expressed on circulating memory T cells (61). The ligand for CCR8, CCL1, is preferentially expressed in human skin, and keratinocyte-derived prostaglandin E_2_ and vitamin D3 can induce CCR8 expression by CD8^+^ T cells, suggesting that it may involve in T_RM_ localization in skin (62, 63). However, the role of CCR8 is currently unclear, since T cells lacking CCR8 can migrate and are maintained in the skin as usual in mouse epidermis following viral skin infection (55). Collectively, CCR10 (53, 64), CCR4 (58), CCR8 (60, 62), and CXCR3 (39) enable memory T cells to migrate to the skin, CLA allowing them to enter the skin (1), and CCR10 and CXCR6 (55) contribute to T_RM_ formation in the skin.

### Retention Mechanisms of Skin T_RM_ Cells

**Figure 2:**
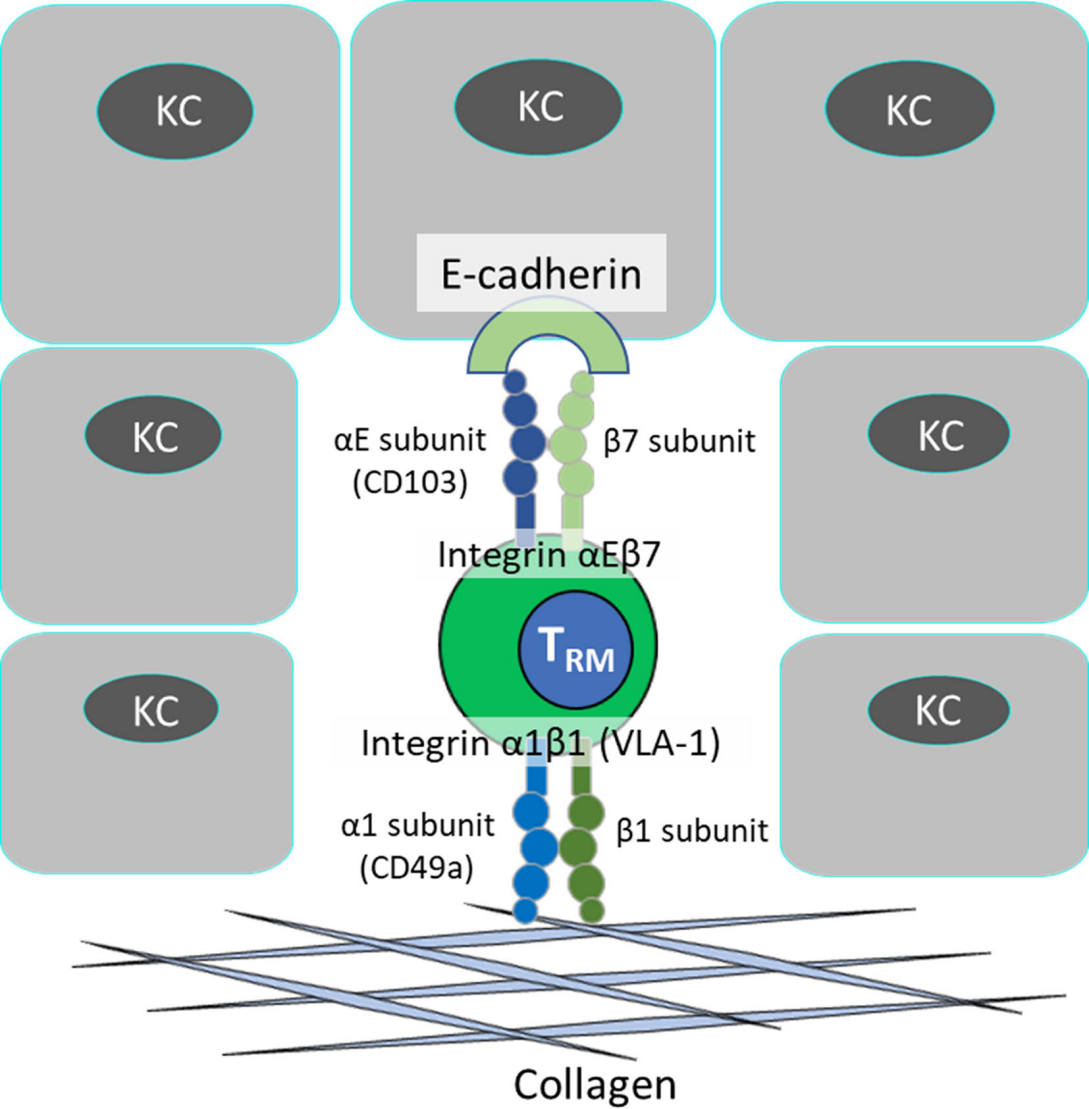
Adhesion of T_RM_ cell in the skin.

The retention properties of skin T_RM_ cells have been widely explored in a murine model. The most recognized markers of skin T_RM_ cells in both humans and mice are CD103 and CD69, which are responsible for T_RM_ retention (65). CD103 is an α-chain of the integrin αEβ7and binds to E-cadherin expressed by keratinocytes (**Figure 2**) and is the most common and widely accepted T_RM_ marker. CD103 expression on CD8^+^ T_RM_ is dependent on the TGF-β (39, 66), which is activated by keratinocyte integrins αvβ6 or αvβ8 (67). In mice lacking this keratinocyte-integrin, T_RM_ cells are unable to express CD103 and cannot persist long term in epidermis (67). CD103 on CD8 T_RM_ cells mediate cell adhesion to the epidermis and thus promote local retention (55). Similarly, CD103^-/-^ CD8^+^ T cells can enter the epidermis but unable to persist long term in the skin as T_RM_ cells (39, 55). TGF-β induces CD103 expression on activated CD8^+^ T cells, but not CD4^+^ T cells, and leads to CD103-mediated adhesion of CD8^+^ T cells, but not CD4^+^ T cells, to monolayer human keratinocyte cultures (68). This may explain the reason why CD4^+^CD103^+^ T cells can exit in the skin, but CD8^+^CD103^+^ T_RM_ cells cannot. However, another study showed that TGF-β also induces CD103 expression on CD4^+^ T cells and mediates cell adhesion to keratinocyte (14). This discrepancy is possibly due to different experimental setups and T cell stimulation methods, and further studies are needed to confirm the function of CD103 on CD4^+^ T cells. Indeed, CD4^+^CD103^+^ cells can be found in human circulation but not CD8^+^CD103^+^ cells (69). Moreover, CD69 expression is very dynamic and can be easily induced *in vitro* upon stimulation (70). By using qPCR, the expression of TGF-β in psoriatic skin is comparable to normal skin, implying that increment of CD103^+^ T cells in psoriasis does not stem from general upregulation of TGF-β expression (68). In tumor context, the interaction between αE(CD103)β7 on tissue-infiltrating lymphocytes and E-cadherin on tumor cells induces cytolytic granule polarization and subsequent exocytosis, leading to tumor cell lysis (71). This suggests that CD103 also exerts some biological activity in addition to the adhesion property.

CD69 is involved in the residency status of T_RM_ cells by downregulating sphingosine 1 phosphate receptor (S1PR1)-mediated tissue egress (72, 73). The vast majority of skin T_RM_ cells in both mice and humans express CD69 (14, 39, 74). The induction of CD69 expression is strongly influenced by antigen stimulation and exposure to pro-inflammatory mediators (72). CD69 is upregulated shortly after memory T cells reaching the skin and CD69 expression is critical for early T cell retention rather than recruitment of T cell into skin (39, 72). However, a recent parabiosis study demonstrated that CD69 expression is inadequate to define a stable residence (27).

α1(CD49a)β1 integrin is one of the T cell receptors for collagen IV, originally termed as Very Late Antigen (VLA)-1. CD49a is upregulated following T cell activation and can be found on circulating T cells before they enter into the skin (75). CD49a-expressing CD8^+^ T cells are enriched in the epidermis of human and mouse skin (8, 37). In an HSV infection mouse model, CD49a increased T_RM_ effector function and promoted T_RM_ persistence in the skin, but not required for CD8^+^ T cell to entry into the epidermis (75). In contrast, in the xenotransplantation model of psoriasis, blocking CD49a inhibits T cell migration into the epidermis, resulting in a decrease of T_RM_ cells and prevention of psoriasis development (76). IL-12 and TGF-β can upregulate CD49a expression on CD8^+^ T cells (75). Not only CD8^+^ T_RM_ cells but also CD4^+^ memory T cells poised for Interferin (IFN)-γ production preferentially express CD49a in human (74, 77). Since IL-12 can induce IFN-γ production and CD49a expression, it is tempting to speculate that in the psoriasis context, IL-17A-producing T_RM_ cells, which preferentially express IL-23R (74), downregulate their CD49a due to a greater influence of IL-23 over IL-12.

Collectively, CD69 is critical for initial formation of T_RM_ cells shortly after T cells enter in the skin, while CD103 is required for T cell adhesion and long-term retention of T_RM_ cells. Ultimately, both CD69 and CD103 are required for T_RM_ formation in the skin. In addition, CD49a regulate the persistence, morphology and effector function of CD8^+^ T_RM_ cells in the skin.

### Characteristics of CD4^+^ Skin T_RM_ Cells

Compared with CD8^+^ skin T_RM_ cells, the characteristics and behavior of CD4^+^ skin T_RM_ cells have been less understood, and probably, they are quite different between mice and humans and remain controversial. In human skin, CD4^+^ T cells can be found in both epidermal and dermal compartments (14), whereas CD4^+^ T cells in murine skin are predominantly in the dermis. In fact, human skin has a thicker epithelial layer and lower density of hair follicles that are crucial for residency of CD4^+^ T_RM_ in mouse skin (78, 79).

Earlier studies showed that the motility of skin-infiltrating CD4^+^ T cells are higher than that of CD8^+^ T cells, and they equilibrate with circulating T cell pool at steady state (78, 80). Skin CD4^+^ memory T cells preferentially accumulate around the hair follicle isthmus and constantly move back and forth to the circulation (78). After cutaneous HSV infection, two distinct HSV-specific memory T cell subsets were found in the skin; the slow-moving CD8^+^ T cell population resided in the epidermis, particularly at the site of infection, whereas dynamic CD4^+^ T cell population rapidly trafficked through the dermis and showed recirculation pattern (80). Indeed, we have previously demonstrated a substantial recirculation of CD4^+^ T cells in the skin to the draining lymph nodes, using a photo-convertible system of Kaede-transgenic mice (81).

A recent study using mice parabiosis experiment identified the CD4^+^ T_RM_ population with prolonged residency in non-lymphoid tissue, which was separated from the circulation and shared transcriptional signatures with CD8^+^ T_RM_ cells. However, this study showed only a limited period of 4 weeks of the extent of residency (82), because the prolonged parabiosis was associated with great equilibration for skin CD4^+^ T cells (78). Another study using alemtuzumab, an antibody targeting CD52 and depleting circulating T cells, showed that CD4^+^CD69^+^CD103^+^ and CD4^+^CD69^+^CD103^-^ persist in the skin without replenishment of the circulating compartment, suggesting that they are T_RM_ populations. Similarly, in *in vivo* studies, CD4^+^CD69^+^CD103^+^ T cells possibly represented a non-migrating resident CD4^+^ T cell population in the dermis (12, 83). However, the dynamic observation of CD4^+^ T_RM_ cells in the skin, particularly in human, is technically challenging, and their migratory behavior cannot be excluded. In contrast, the xenografting model with human skin showed that CD4^+^CLA^+^CD103^+^ T_RM_ cells down-regulate CD69 expression, exit from the skin, and reach into the circulation (69). These cells in the blood and skin are clonally related and share their function and transcriptional profiles. CD4^+^ T_RM_ cells were reported to play a role against skin infection with *L. major* (13) and *C. albicans* (12). Recently, resident memory Th2 cells in the lung exhibit a distinct CD4 population and play a critical role in an allergic asthma murine model (19). Furthermore, in experimental colitis, CD4^+^ T_RM_ cells play a crucial role in the regulation of intestinal inflammation, and they were found in the colon of inflammatory bowel disease patients (16). These studies support the existence and critical role of CD4 T_RM_ cells in tissue-specific immune and inflammatory diseases.

Originally, T_RM_ cell was defined as a memory T cell population that persists long-term in peripheral tissue and do not migrate back to the circulation. According to this definition, not all skin-infiltrating T cells are resident memory T cells. There are only a fraction of these cells that represent the authentic T_RM_ population. A similar definition may be applied to CD4^+^ T_RM_ cells. In fact, the residence is difficult to quantify, and there are no perfect markers to define a permanent resident T cell. CD103 and/or CD69 may not be sufficient for defining the residence status of skin infiltrating T cells, especially CD4^+^ T cells (14, 84). Collectively, it is tempting to postulate that CD4^+^ T_RM_ cells are generally more dynamic and have a distinct migratory behavior compared to CD8^+^ T_RM_ cells in human skin. Meanwhile, in some inflammation or infection context, CD4+ T_RM_ cells play a crucial role and may persist in the skin for an extended period.

## Development of Skin T_RM_ Cells

A different subset of memory CD8^+^ T cells contribute to an immune memory response in different aspects and locations. Once naïve CD8^+^ T cells are activated, they differentiate into pooled effector CD8^+^ T cell populations, which are composed of SLECs and MPECs. MPECs are characterized by CD127^hi^KLRG1^lo^ populations, while SLECs are KLRG1^hi^ populations. After clearance of inflammation or infection, the majority of SLECs undergo apoptosis, whereas MPECs turns into a heterogeneous subset of memory T cells (85). Historically, memory T cells were divided into central memory (T_CM_) cells that express high lymphoid homing molecules and recirculate through SLOs, and effector memory T (T_EM_) cells that lack lymphoid homing molecules (86). From the current literature, memory T cells can be broadly divided into four main populations in the murine model. (1) T_CM_: expressing lymph node (LN) homing molecules (CCR7^+^CD62L^+^CX3CR1^-^) and mainly surveying SLOs. (2) T_EM_: expressing CCR7^-^CD62L^-^CX3CR1^+^ and predominantly surveying the blood. (3) peripheral memory T cells (T_PM_): expressing CCR7^+^CD62L^-^CX3CR1^int^ and preferentially patrolling peripheral tissues and migrate to blood and LN. (4) T_RM_: persisting for a long term in peripheral tissues.

By immunizing mice with a protein antigen, chemical hapten, or non-replicating virus, T_RM_ cells from the treated skin and distant skin as well as the draining and distant LNs contain identical TCR cells in both T_RM_ and T_CM_ compartment, suggesting that T_RM_ and T_CM_ cells may be derived from common naïve T cell precursors (87). However, equal contribution of individual naïve clones to formation of T_RM_ subsets has not been definite. Using a lineage-tracing technique to track individual naïve CD8^+^ T cells responding to skin vaccination, it was shown that individual T cell clones contribute differentially to the formation of T_RM_-poised effector T cell subset, which has a capacity to subsequently form T_RM_ population (88). The propensity to form T_RM_ populations is disparately distributed over T cell clones, implying that this fate must be committed before clonal expansion. The heterogeneity of circulating vaccine-specific effector T cell pool can be divided into four distinct populations based on the gene expression profiles, including effector cell, intermediate cell, circulating memory T cell-like precursor, and T_RM_-like precursor. This study revealed the existence of T_RM_ cell precursor in circulation and their commitment to T_RM_ cells before entering into the skin (88).

**Figure 3:**
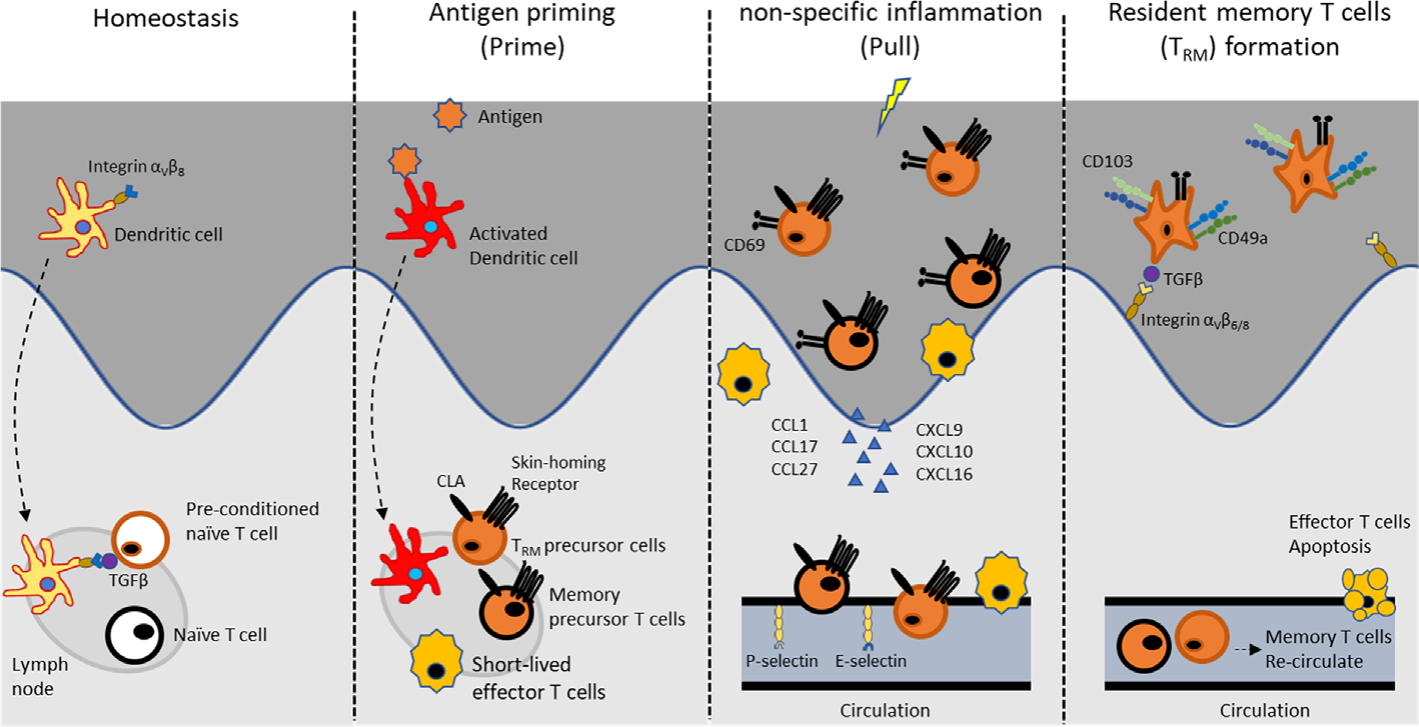
Development of skin T_RM_ cell.

The existence of pre-commitment T_RM_ cells in circulation was further supported by an elegance study on the role of dendritic cell in T_RM_ cell formation (89) (**Figure 3**). This study revealed that the formation of skin T_RM_ cells requires interaction between naïve CD8^+^ T cells and migratory dendritic cells (DCs) from the skin at a steady state. This process depended on the presence of TGF-β, which activates V-integrins on migratory DCs. In fact, lack of V-integrins on CD11c^+^ DCs resulted in a substantial reduction in epidermal CD8^+^ T cells, but did not affect dermal CD8^+^ T cells or other skin immune populations. The expression of a V-integrins on DCs during immune homeostasis, but not in priming state, was required for pre-conditioning naïve CD8^+^ T cells for effective T_RM_ cells formation (89). Therefore, T_RM_ fate decisions on T cells seem to happen earlier than expected, and this event appears to be controlled primarily by a cross-talk between local skin and draining LNs *via* DCs. Indeed, DCs are able to instruct T cells to migrate to a specific location. For example, DCs in skin-draining LNs and mesenteric LNs induce the expression of tissue homing molecule that elicits tropism for skin and gut, respectively (90, 91). Earlier studies showed that individual naïve T cells contribute differentially to short-term effector cells and long-term memory cells, and the fate of each naïve T cells is unpredictable (92). However, the subsequent study revealed the clonal bias of T_RM_ precursors within heterogenous memory populations (88).

Non-specific inflammation is sufficient to attract CD8^+^ T cells into the inflamed tissue and adopt T_RM_ cells in the skin (93, 94), suggesting that T_RM_ cells in the skin do not require cognate antigen for their establishment. Basically, the skin immune cells respond to an invader such as hapten and secrete pro-inflammatory cytokines that induce dendritic cell migration and maturation (95). Endothelial cells increase the expression of adhesion molecules; CD54 (ICAM-1) and CD106 (VCAM-1), which guide T cell entry into the tissue. In addition, chemokines, Chemokine ligand (CCL)2 to 5, CXCL9, and CXCL10 are secreted from keratinocyte and innate immune cells, and this initial step is induced by a non-specific inflammation process and is a fundamental mechanism to recruit T cells into inflamed skin (96). However, the presence of cognate antigens enhances T_RM_ cell formation. Moreover, antigen challenges at the skin lead to generalized seeding of antigen-specific T_RM_ cells, which are found at the highest density at sensitizing area (39, 87).

## Maintenance of Skin T_RM_ Cells

**Figure 4:**
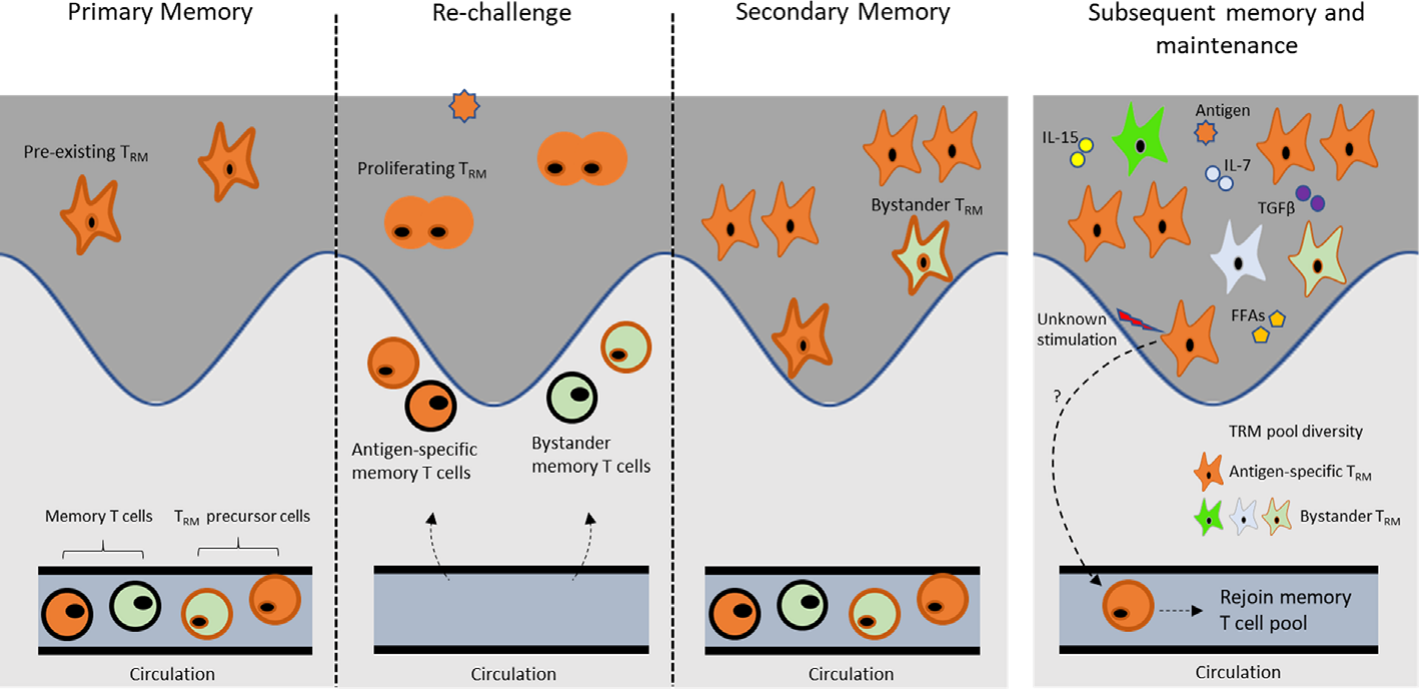
Maintenance of skin T_RM_ cell.

A whole-genome bisulfate sequencing study suggests that T_RM_ cells have a high plasticity and a development potential comparable to T_CM_ and T_EM_ cells, indicating that they are not terminally differentiated (97). In addition, T_RM_ cells can proliferate *in situ* in response to viral challenge, further supporting their as yet undifferentiated status (94). Different factors are required for maintenance of T_RM_ cells, depending on individual tissues (98). Skin CD8^+^ T_RM_ cells can be maintained in the skin for a long period (65, 87). Several factors, including local antigens, cytokines, and metabolites, contribute to T_RM_ maintenance (**Figure 4**). A disparate level of skin residency may exist in skin T_RM_ cells. While certain subsets of skin T_RM_ cells have long-term residency, other subsets transiently reside in the skin and possibly migrate out to the circulation.

### Effects of Cognate Antigens

Although local antigen is not required for skin recruitment of circulating CD8^+^ T cells to obtain the T_RM_ phenotype, antigen exposure greatly amplifies the number of CD8^+^ T_RM_ cells (99). Local antigenic challenge induces antigen-specific T_RM_ cell proliferation, and they are maintained as epidermal T_RM_ pool (94). Intriguingly, the subsequent pool of T_RM_ cells after antigen reencounter is generated mainly from the pre-existing T_RM_ cell population, rather than from circulating memory T cell compartment (94, 100). A self-sustained capacity of T_RM_ cells in the skin seems to be independent of CD4^+^ helper T cells and CD11c^+^ cells (100). The contribution of circulating memory T cells in the local immune response may depend on the density of the pre-existing T_RM_ population, suggesting the flexibility of circulating T_CM_ cells to support T_RM_ population. Moreover, even with the newly seeded, unrelated T_RM_ population in the skin, the number of pre-existing T_RM_ cells remain largely unchanged. Initial activation of skin T_RM_ cells requires antigen recognition, which represents T_RM_-mediated skin protection and is ultimately changed to an antigen independent reaction (101). T_RM_ cells thus exert a protection capacity, depending on their local density in skin (94). A question arises as to how local antigen influences composition of skin T_RM_ cells from a pool of polyclonal skin-infiltrating memory precursors during active infection or inflammation. It has been revealed that local antigen-dependent cross-competition contributes to shaping the polyclonal T_RM_ cell repertoire in the skin, whereas this event is not observed in SLOs (102). Therefore, the local antigen-dependent self-amplification and cross-competition processes may serve as a mechanism to modulate local T_RM_ composition in response to a variety of invaders and responsible for maintenance of T_RM_ cell population in skin.

### Fatty Acids for the Maintenance of Skin T_RM_ Cells

One of the basic needs for life is food. The skin has a unique microenvironment where lipids are rich even with shortage of nutrients. Skin T_RM_ cells reside in the epidermis, and thus, they are relatively independent from blood circulation. Although nutrients may diffuse from the dermis to the epidermis, the local energy source seems to be required for T_RM_ cells. Fatty acid binding proteins (FABPs) are a group of intracellular molecules that mediate lipid trafficking and metabolism (103). FABPs originally consist of adipose FABP (A-FABP) and epidermal FABP, which encoded by *Fabp5.* E-FABP is expressed on keratinocytes and immune cells, including T cells and macrophages (104). High-fat diet upregulated E-FABP expression and promote skin inflammation, suggesting the role of lipid metabolism in immune regulation (105). Recently, it was shown that CD8^+^ T_RM_ cells utilize exogenous lipids in the skin as an energy source for their survival. T cells lacking Fabp4 and Fabp5 cannot uptake and utilize exogenous free fatty acid (FFA), which results in a reduction of long-term survival and impaired functional properties of CD8^+^ T_RM_ cells *in vivo*. This deficiency has no effect on T_CM_ cell survival. Interestingly, the significance of lipid metabolism for T_RM_ survival is increased over time, suggesting metabolic adaptation to the skin environment. It is proposed that CD8^+^ T_RM_ cells utilize local lipid as an energy source to maintain their functional competence and longevity in the skin. Similarly, CD8^+^ T_RM_ cells in the skin also increase the expression of FABP4 and FABP5 (106). It seems that the impact of FABP deficiency is not only limited to CD8^+^ T_RM_ cells but also affects CD4^+^ T cells and DCs. Upregulation of FABPs on CD4^+^ T cells promotes IL-17 expression, while the loss of FABPs is associated with enhanced expression of FoxP3 (104), suggesting the role of E-FABP and Th17/Treg balancing. In addition, FABP-deficient mice showed an altered antigen-presenting function of dendritic cells and macrophages (107). The limitation of energy resources in the epidermal niche possibly influences the T_RM_ cell density and survival. A recent study demonstrated that CD8^+^ T_RM_ cells displace pre-existing dendritic epidermal T cells (DETCs) from the epidermis because they have a superior metabolic fitness (108).

### Cytokines

Despite the likeness between IL-15 and IL-2, including shared receptor subunit, IL-15 has a perceptible difference in immunomodulatory properties (109). Basically, IL-15 promotes proliferation and survival of circulating memory CD8^+^ T cells but did not affect regulatory T cell populations in human (110, 111). IL-15 deficient mice showed a reduction of CD8^+^ T_RM_ cell number (39, 112) but slightly increased CD4^+^ T_RM_ cells in the skin, while the numbers of CD8^+^ T cells and CD4^+^ T cells in SLOs were not different between IL-15-deficient and WT mice (112). Keratinocytes at hair follicle has been shown as the main source of IL-15 for maintaining CD8^+^ T_RM_ cells in the skin. In addition to IL-15, IL-7 from hair follicle also influence on both CD8^+^ T_RM_ and CD4^+^ T_RM_ cells persistent in the skin. However, the requirement of IL-15 for T_RM_ maintenance may vary depending on the tissue and context of inflammation (41). Apart from maintenance property, IL-15 strongly induces perforin and granzyme B expression in CD8^+^CD103^+^CD49a^+^ T_RM_ cells but not in CD8^+^CD103^+^CD49a^-^ T_RM_ cells isolated from normal human skin (74). TGF-β is a pleiotropic cytokine that is produced in an inactive form that requires specific integrins on keratinocyte to activate them (113). Activated-TGF-β induces CD8^+^ T_RM_ cells to express CD103, which is mandatory for their retention and long-term persistence in the skin (39, 55). Collectively, keratinocytes play an important role in establishing long-term T_RM_ cell populations by providing local mediators like IL-15, IL-7, and activated TGF-β.

## Skin T_RM_ Cells in Cutaneous Defense System Against Pathogens

Although the pathophysiological roles of skin T_RM_ cells encompass several aspects (65), they serve primarily as a critical component of cutaneous immune defense. T_RM_ cells act as peripheral sentinels providing rapid immune response against invading pathogens (114). Infection with pathogenic microorganisms leads to directed homing of T cells to the appropriate tissues, such as the skin. Subsequently, most antigen-specific memory T cells reside in the non-lymphoid organs, convey tissue-resident memory, and mount durable protective immunity in the skin.

Virus is a major pathogen to which skin T_RM_ cells respond, and a number of valuable findings have been obtained from studies on virus infection. T_RM_ cells can autonomously regulate the local T_RM_ composition to mediate immunosurveillance independently of circulating memory T cells (94, 100). Skin T_RM_ cells are activated and proliferate *in situ* upon encounter with virus-infected cells, and do not migrate out of the skin. As a consequence, secondary T_RM_ cell populations were mainly derived from pre-existing T_RM_ cell populations and the precursors recruited from the circulation. In subsequent infections, the pre-existing skin T_RM_ cell populations are not displaced by the newly generated T_RM_ cells, enabling multiple T_RM_ cell specificities to maintain a diverse immune response within the tissue (94). Consistently, mucosal T_RM_ cells are highly motile, but pause and undergo *in situ* division after local antigen challenge. T_RM_ cell reactivation triggers the recruitment of recirculating memory T cells that undergo antigen-independent T_RM_ cell differentiation *in situ*. The proliferation of pre-existing T_RM_ cells dominates the local mucosal recall response and contribute most substantially to the boosted secondary T_RM_ cell population (100).

CD8^+^ T_RM_ cells seem to play a major role in cutaneous defense against virus. After resolution of skin vaccinia virus infection, antigen-specific circulating memory CD8^+^ T cells migrate into the skin. Memory T cells that reside at these surfaces provide a first line of defense against subsequent infection (6, 115, 116).

The local cytokine environment within the skin determines the differentiation state and persistence of the central and peripheral memory-T-cell pool (67). CD8^+^CD103^+^ T_RM_ cells develop in the skin from epithelium-infiltrating precursor cells that lack expression of the effector-cell marker. Following the entry of the T cells into the epidermis, the local mediators such as IL-15 and transforming growth factor (TGF)-β are required for the formation of long-lived T_RM_ cell population in skin (39). The retention of tissue-resident memory T cells is mediated by TGF-β, which up-regulates CD103 expression and down-regulates CCR7 expression. Besides microbial pathogens, topical application of chemical allergen to skin causes delayed-type hypersensitivity and amplifies the number of antigen-specific CD8^+^ T_RM_ cells at challenged site (117). Expanded T_RM_ CD8^+^ T cells in the skin are derived from memory T cells recruited out of the circulation. Expanded T_RM_ CD8^+^ T cells significantly increase anti-viral protection.

In addition to CD8^+^ cells, CD4^+^ T_RM_ cells are also involved in microbial defense. CD4^+^ T_RM_ cells play a role in cutaneous fungal infection (12). *Candida albicans* (*C. albicans*) is a common dimorphic fungal pathogen to which human subjects are exposed early in life, and by adulthood. In a *C. albicans* infection mouse model, dermal γδ T cells producing IL-17 are the main effector cells in the initial infection, and then, αβTh17 effector T cells become predominant. By day 30 after infection, the CD4^+^ T_RM_ cells become the main population of IL-17-producing T cells that react to *C. albicans*. Between 30 and 90 days after infection, these reactive CD4^+^ T cells acquire expression of CD69 and CD103, the retention markers, and reside in the papillary dermis. These T_RM_ cells are more effective to eradicate *C. albicans* than recirculating T cells (12).

Recently, the preclinical studies on T_RM_-targeted vaccination have shown a favorable outcome. Intranasal (118) and mucosal (119) administration of vaccine generated protective T_RM_ cells in the lung and airway of mice. Direct vaccination (118, 119) or delivery vaccine vectors to a specific tissue (120, 121), rather than parenteral route, generated antigen-specific T_RM_ cells, thereby mediating effective protection independent of circulating memory T cells. In addition, a “prime and pull” strategy (122), which combines vaccination with local application of chemokines, effectively generated T_RM_ cells. These studies suggest that protective T_RM_ cells can be generated through vaccination, especially tissue-targeted approaches that give a better protection than ordinary parenteral route. Since the skin is an accessible tissue for administration of vaccine, a question arises whether immunization through the skin can generate T_RM_ cells in other organs or barrier tissues. In fact, the smallpox vaccine, which is one of the most effective vaccine in history, was delivered by skin scarification (123). In a murine model, the localized virus skin infection (35) or skin immunization (87) can generate antigen-reactive T_CM_ cells and skin T_RM_ cells that reside within the entire skin and possibly in the lung (124). Besides, the combination of “prime and pull” with a prime boost approach was reported to be very effective to produce protective T_RM_ cells (125). These suggest the possible role of the skin as a T_RM_-targeted vaccination strategy. Further understanding of how skin dendritic cells shape the T_RM_ precursor pool (89), which have a potential to transform into tissue-specific T_RM_ cells, may provide a crucial information for the development of T_RM_-targeted vaccination. Furthermore, skin resident memory T cells also play a protective role in skin infection, such as HSV (35), *C. albicans* (12), leishmania major (13), and in skin cancers, such as melanoma (126) and squamous cell carcinoma (127). They also play a pathogenic role in some autoinflammatory skin diseases; vitiligo (9, 128), psoriasis (8) and alopecia areata (10). Thus, the vaccination-induced T_RM_ cell strategy may also have a potential to become a novel therapeutic approach to protect the skin from infection, prevent tumor growth, or suppress autoreactive immune responses.

## Skin T_RM_ Cells in Psoriasis

**Figure 5:**
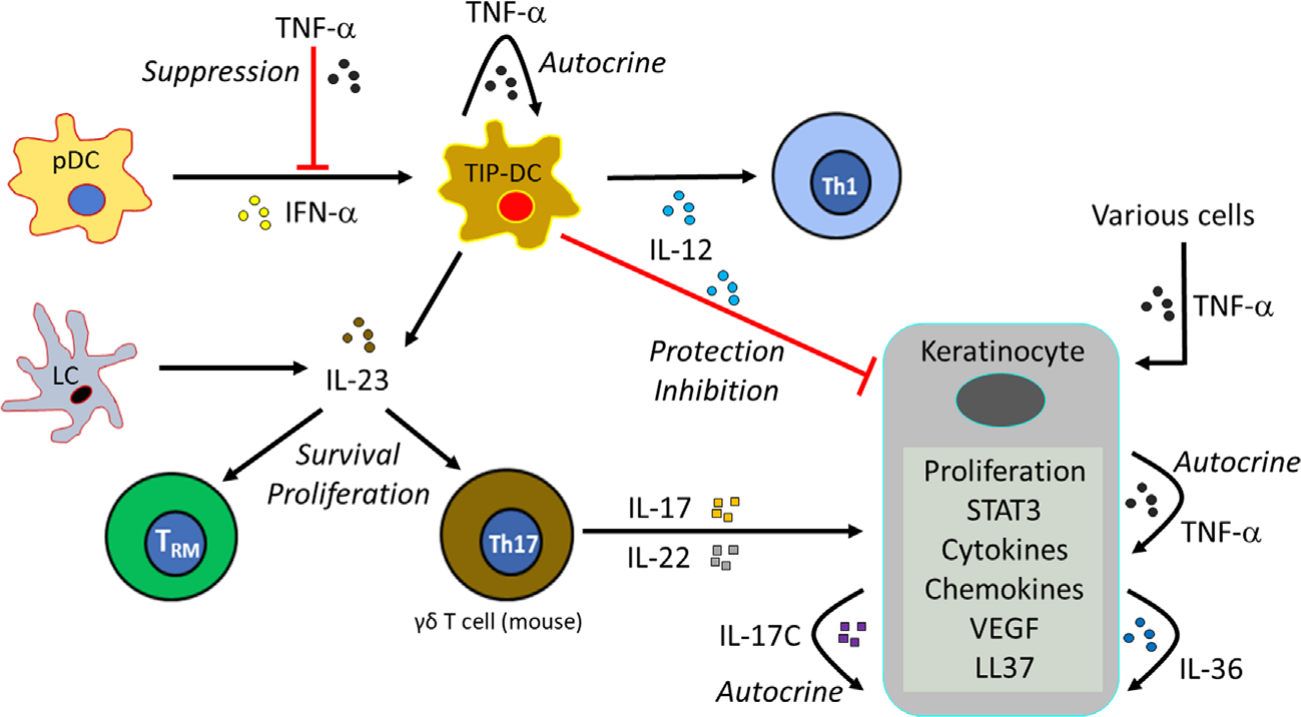
Mechanism of psoriasis.

Psoriasis is a common chronic inflammatory skin disease, and the pathogenesis underlying psoriasis has been extensively studied (**Figure 5**). CD4^+^ T cells producing interleukin (IL)-17, named Th17 cells, play an essential role in its pathogenesis (129). Th17-derived cytokines, IL-17A, IL-17F and IL-22, induce epidermal acanthosis, which represents an intriguing histological finding of psoriasis and results from the proliferation of epidermal keratinocytes. These mediators stimulate keratinocytes to produce TNF-α, IL-8, and vascular endothelial growth factor, thereby promoting inflammation, neutrophil recruitment, and angiogenesis (129). For maintenance of Th17 cells, IL-23 is required and secreted from inflammatory DCs or TNF-α and iNOS-producing DCs (TIP-DCs) Psoriasis and other Th17-mediated skin diseases (129). Epidermal Langerhans cells are another source of IL-23 in a certain condition (130). Keratinocytes are also activated by their own cytokines, such as IL-17C, IL-36, and TNF-α, in an autocrine manner (131, 132). In addition, antimicrobial peptides released from keratinocytes and (IFN)-α from plasmacytoid DCs has been considered to play initiative roles for the development of psoriatic lesions (133). Meanwhile, a self-regulatory autocrine mechanism is disturbed in epidermal keratinocytes of psoriasis patients (134).

The cytokine network in psoriasis has been proven by the therapeutic effectiveness of biologic antibodies that block individual cytokines, including TNF-α, IL-23/IL-12p40, anti-IL-23p19, IL-17A, and IL-17 receptor (135). Although biological drugs are effective, there are variations in the responsiveness between patients (136). Moreover, upon withdrawal of the biologics, the skin lesions often recur. Psoriasis plaques are seen in a recurrent manner especially at the originally affected sites (137). Thus, even after clearance of skin lesions, some immunocompetent cells possibly remain in the previously affected, currently normal-appearing skin. A number of studies have suggested the pathogenetic role of skin T_RM_ cells in psoriasis (8, 74), particularly as a strong candidate that evokes recurrence (2). Notably, T_RM_ cells in psoriatic skin can produce certain cytokines and decreased in number after improvement (74). CD8^+^ T_RM_ cells reside even in disease-naïve, non-lesional sites of psoriasis patients possibly in correlation with disease duration (138).

**Figure 6:**
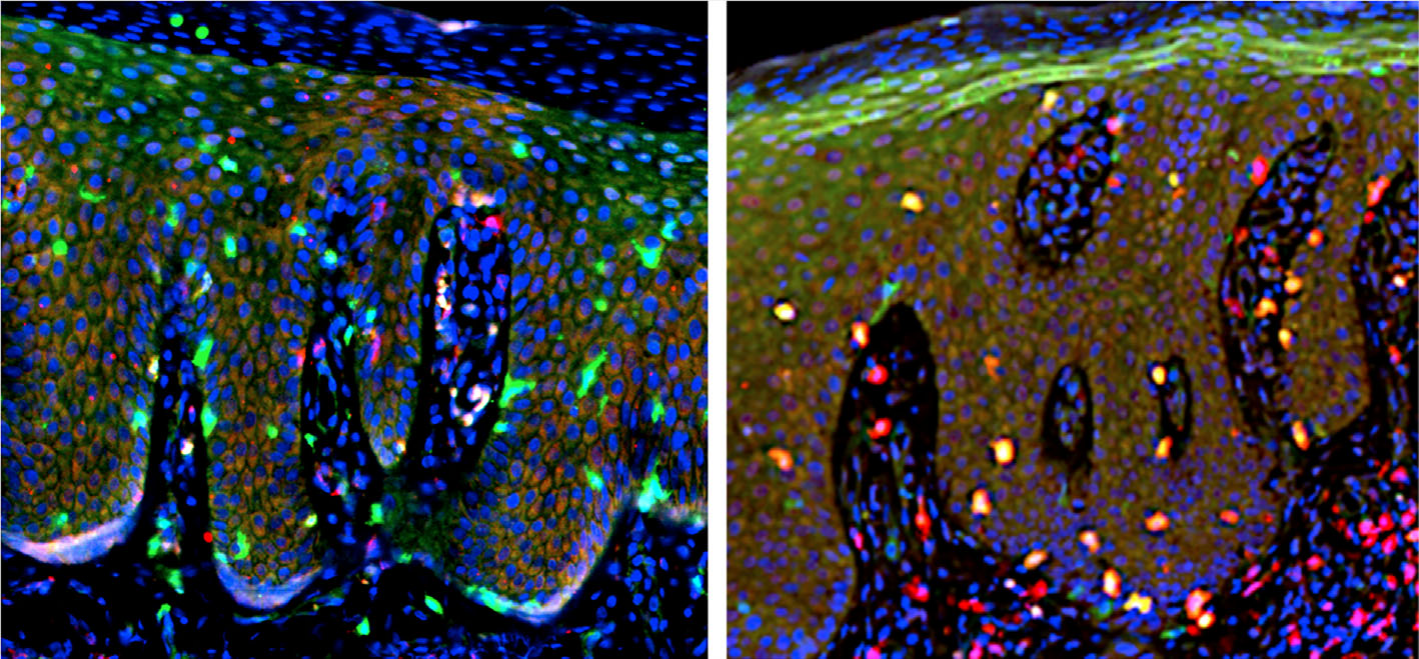
Double immunofluorescent staining. Left: CD4 (red) and CD103 (green). Right: CD8 (red) and CD103 (green). Merged yellow color (right) indicate cells positive for both CD8 and CD103, representing T_RM_ cells.

The skin T_RM_ cells are positive for tissue-retention markers CD103 and CD69, but negative for lymphoid homing markers CD62L and CCR7 (139). Double immunofluorescent staining for CD3, CD4, or CD8 (red) along with CD103 (green) is shown, and the merged yellow color represents cells positive for both (**Figure 6**). CD3^+^ T cells infiltrate into both epidermis and dermis, and majority of the T cells in the epidermis co-expressed CD103. CD4^+^ cells mainly infiltrate in the dermis and scarcely express CD103. CD8^+^ cells infiltrating in the epidermis are positive for CD103, while those in the dermis were mostly CD103^-^. Thus, the majority of epidermal T cells are CD8^+^CD103^+^ T_RM_ cells and a small number of CD4^+^CD103^+^ T_RM_ cells infiltrate in the dermis. A few CD8^+^CD103^+^ T_RM_ cells are present in the papillary and subpapillary layers. The number of CD8^+^CD103^+^ T_RM_ cells in the epidermis tends to correlate with the epidermal thickness (70), suggesting the role of T_RM_ cells in the formation of psoriatic lesions.

When CD103^+^, CD103^-^, CD69^+^, and CD69^-^ T cells were isolated and expanded *ex vivo* with anti-CD3/CD28 Ab and IL-2 (140–142), the positive and negative expression of CD103 was unchanged (70). However, CD69 expression can be changed bidirectionally by cultivation, suggesting the unsteady, fluctuated expression of CD69. By using skin-derived, *ex vivo* expanded T cells (140–142), we conducted to characterize the cytokine profile of CD103^+^ skin T_RM_ cells, especially, epidermal CD8^+^CD103^+^ T_RM_ cells (39, 74). In T cell samples expanded from psoriasis lesional skin, a part of CD8^+^ T cells co-expressed CD103, and this CD8^+^CD103^+^ T cells are considered to be epidermal T_RM_ cells. CD4^+^CD103^+^ cells are present at a much lower frequency. CD103^+^ T cells were mostly CD8^+^CD45RO^+^CD45RA^-^CD69^+^ memory T cells with a skin-homing potential, i.e., partially CCR6^+^ and mostly CCR7^-^CD62L^-^. They contained both CXCR3^+^CD49a^+^ and CXCR3^-^CD49a^-^ populations. These findings are in accordance with the importance of CD8^+^ T cells in psoriasis pathogenesis (138, 143–145).

The cytokine production pattern of skin T_RM_ cells has been a crucial issue, because their function is generally determined by the released cytokines. Skin T_RM_ cells remain longer in the same position than effector memory T cells (51) and produce certain cytokines in relation to psoriatic etiology (39, 74, 146). CD103^+^ T_RM_ cells produce IFN-γ, IL-17A, and IL-22 (39, 74, 147). In the *ex vivo* expanded T cells, certain populations of CD8^+^CD103^+^ T cells produce IFN- γ, IL-17A or IL-22, while CD4^+^CD103^+^ T cells scarcely elaborate these cytokines. In CD8^+^ T cells, CD103^+^ T_RM_ cells more frequently produce IL-17A than CD103^-^ T cells. Thus, CD8^+^CD103^+^ T_RM_ cells efficiently produce IL-17A.

The sorted CD103^+^ cells expressed CXCR3 or CD49a at a frequency of 28%, sharing the feature with Tc1 or reported IFN-γ-producing T cells (39, 74). The counterpart cells were CD49a negative or low, supposedly corresponding to IL-17A-producing T cells (39, 74). Taken together these observations, CD8^+^CD103^+^ T_RM_ cells can be divided into two types: CD49a^-^IL-17A^+^ and CD49a^+^IFN-γ ^+^ types. It is assumed that the former type is closed associated with psoriasis, while the latter type play a role in vitiligo (74).

Skin T_RM_ cells are associated with not only the development of psoriasis (39, 138, 139), but also its clinical course. T_RM_ cells producing IL-17A in resolved psoriasis epidermis could be associated with early relapse (148), and CD8^+^ T_RM_ cells with IL-17A-producing potential in disease-naïve, non-lesional sites possibly correlate with disease duration (138). Thus, IL-17A-producing CD103^+^ T_RM_ cells may have an influence on the future clinical course of psoriasis. We surveyed the 10 patients as to whether oral cyclosporine, oral phosphodiesterase 4 (PDE4) inhibitor or systemic biologics was initiated within one year after the biopsy. The results showed that the patients having entered these advanced therapies possessed higher frequencies of CD8^+^CD103^+^IL-17A^+^ T_RM_ cells (70). Among CD103^+^ T cells, the frequencies of CD8^+^CD103^+^IL-17A^+^ and CD4^+^CD103^+^IL-17A^+^ cells tended to be higher in the advanced therapy group than in the non-advanced therapy group. The CD8^+^ T_RM_ cells showed a high frequency compared with the CD4^+^ T_RM_ cells. Thus, IL-17A-producing CD8^+^CD103^+^ T_RM_ cells may be associated with a progressive clinical course of psoriasis rather than the severity of skin lesions. One can speculate that upon provocation of the skin with stimulants causing Köbner phenomenon, reactivated CD8^+^CD103^+^ T_RM_ cells initiate the psoriatic condition with IL-17A.

## Skin T_RM_ Cells in Vitiligo

**Figure 7:**
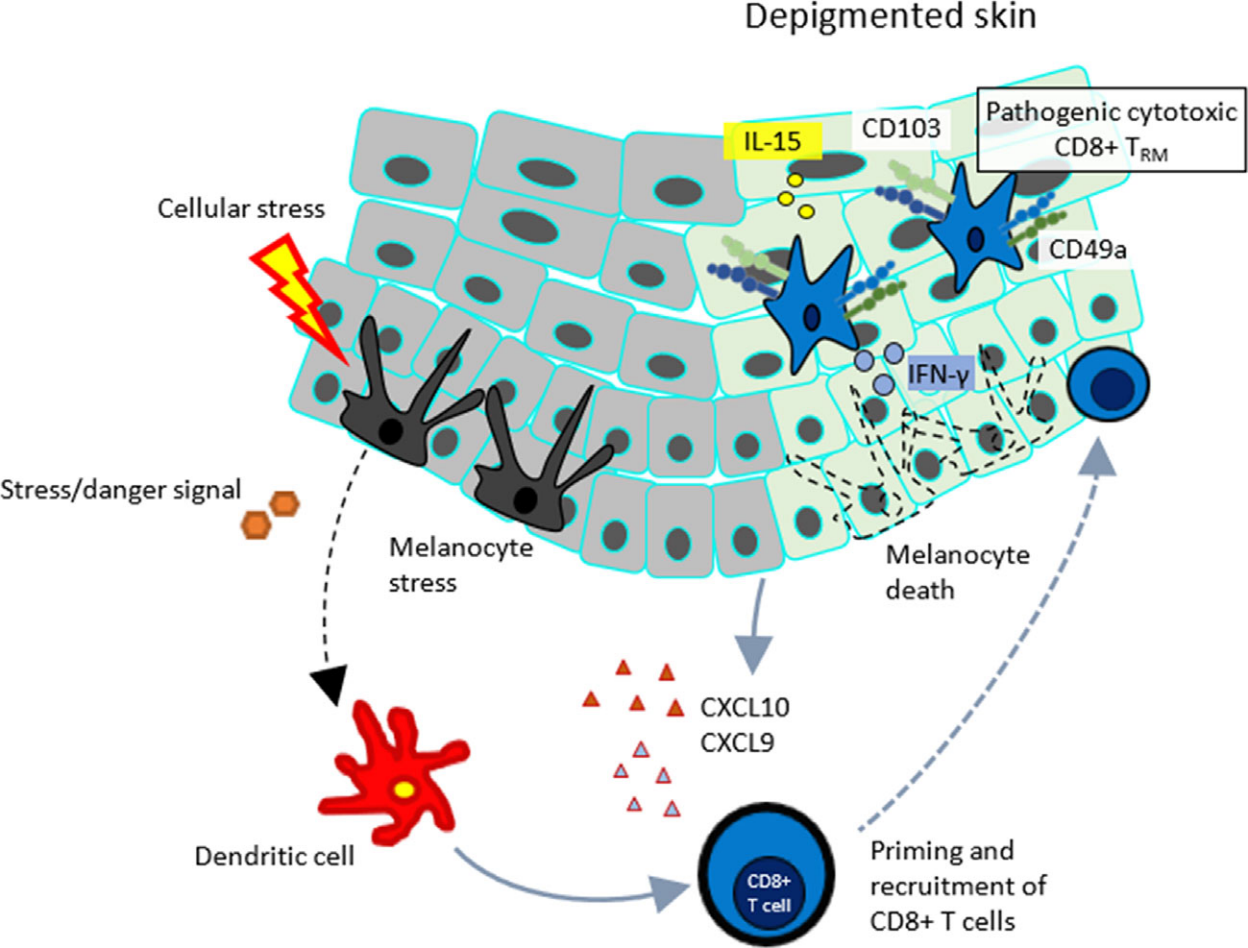
Mechanism of vitiligo.

Vitiligo is an autoimmune skin pigmented disorder mediated by autoreactive IFN-γ- producing CD8^+^ T cells that attack melanocytes, leading to loss of skin pigmentation (**Figure 7**). The appearance of vitiligo in melanoma patients treated with anti-PD-1 immune checkpoint inhibitors is well known as an immune-related adverse event. Autoreactive cytotoxic lymphocytes (CTLs) against normal melanocytes as well as melanoma tumor cells are activated by the antibody therapy (149).

When aberrantly activated, skin T_RM_ cells have a profound role in vitiligo and melanoma (128). CD8^+^CD103^+^CD69^+^CD49a^+^ T_RM_ cells serve as CTLs (74, 143). Accordingly, most of CD8 T_RM_ cells express CXCR3 in vitiligo, indicating inclusion of the population of melanocyte-specific CD8 T cells, which display increased production of IFN-γ and tumor necrosis factor-α with moderate cytotoxic activity (143). Autoreactive T_RM_ cells are also present in mouse models of vitiligo. However, it was found that not only skin T_RM_, but also recirculating memory T cells, plays a role in the development of vitiligo (150). They sense autoantigen in the skin long after stabilization of disease and produce IFN-γ, which further induces CXCL9, and CXCL10 production. Blockade of recirculating memory T cell recruitment to the skin with FTY720 or depletion of them with an antibody reverse disease, indicating that recirculating memory T cells cooperate with T_CM_ to maintain disease (150).

Targeting of T_RM_ cells could become a promising treatment strategy for vitiligo. Moreover, recent evidence demonstrates that induction of melanoma-reactive T_RM_ cells is needed to effectively control tumor growth (9). In a murine model, IL-15 is essential for T_RM_ formation and functions. Both human and mouse T_RM_ cells express IL-15Rβ subunit CD122, and that keratinocytes or other antigen presenting cells up-regulate the expression of IL-15Rα subunit CD215, thereby promoting activation of T cells. Blocking the IL-15 signaling with an anti-CD122 antibody improves the skin depigmentation in mice with established vitiligo. Although prolongation of treatment with anti-CD122 antibody depletes T_RM_ cells from the skin lesion, and the short-term treatment with systemic or local anti-CD122 antibody inhibits IFN-γ production from T_RM_ cells and promotes skin repigmentation (151). Thus, targeting IL-15 signaling *via* CD122 may be a promising therapy for vitiligo.

## Skin T_RM_ Cells in Cutaneous Lymphomas

Cutaneous T-cell lymphoma (CTCL), encompassing mycosis fungoides (MF), Sézary syndrome (SS) and other variants, is a mature T-cell lymphoma, which is currently thought to develop primarily in the skin by a clonal expansion of a transformed, T_RM_ cell (14, 112, 152, 153).

In the epidermis, both CD8^+^CD103^+^ and CD4^+^CD103^+^ T_RM_ are present and have potent effector functions (14), although the former CD8^+^ population is present at a higher frequency in the normal and psoriatic lesional skin (70, 138, 142). Skin T_RM_ in the dermis are CD4^+^CD69^+^CD103^-^. In recirculating T cells, there are CCR7^+^L-selectin^+^ central memory T cells (T_CM_) and CCR7^+^L-selectin^-^ skin-tropic migratory memory T cells (T_MM_). Clonal malignant T cells from the blood of Sézary syndrome (SS) patients universally coexpress CCR7 and L-selectin as well as the differentiation marker CD27, a phenotype consistent with T_CM_ cells (14). CCR4 is also universally expressed at high levels, and there is variable expression of other skin addressins (CCR6, CCR10, and CLA). In contrast, T cells isolated from MF skin lesions lack CCR7/L-selectin and CD27 but strongly express CCR4 and CLA, a phenotype suggestive of skin T_RM_ cells (152). CD4^+^ and CD8^+^ skin T_RM_ cells reside predominantly within the hair follicle epithelium. Hair follicle expression of IL-15 is required for CD8^+^ skin T_RM_ cells, and IL-7 for CD8^+^ and CD4^+^ skin T_RM_ cells, to exert epidermotropism (112).

However, the skin T_RM_ origin concept for the development of MF does not explain the occurrence of multiple, widespread skin lesions. A whole-exome sequencing approach to detect and quantify TCR-α, β, and γ clonotypes in tumor cell clusters suggests the existence of multiple T-cell clones within the tumor cell fraction, with a considerable variation between patients and between lesions from the same patient (153). Thus, circulating neoplastic T-cell clones may continuously replenish the lesions of MF, thus increasing their heterogeneity by a mechanism analogous to the consecutive tumor seeding.

Adult T-cell leukemia/lymphoma (ATLL) is a malignancy of mature T cells caused by human T-cell leukemia virus type I. Approximately 50% of ATLL patients exhibit skin lesions where malignant CD4^+^CD25^+^ T cells histologically show epidermotropism (154). We documented a case of adult T-cell leukemia/lymphoma (chronic type), which had a phenotype of CD4^+^CD25^+^CD69^+^CD103^+^ T_RM_ cells (155), indicating the T_RM_ property of this case and the presence of T_RM_ malignancy in cutaneous lymphomas other than MF. Taken together these observations in CTCL and ATLL, the vast majority of cutaneous lymphomas are derived from skin CD4^+^ T_RM_ cells.

**Figure 8:**
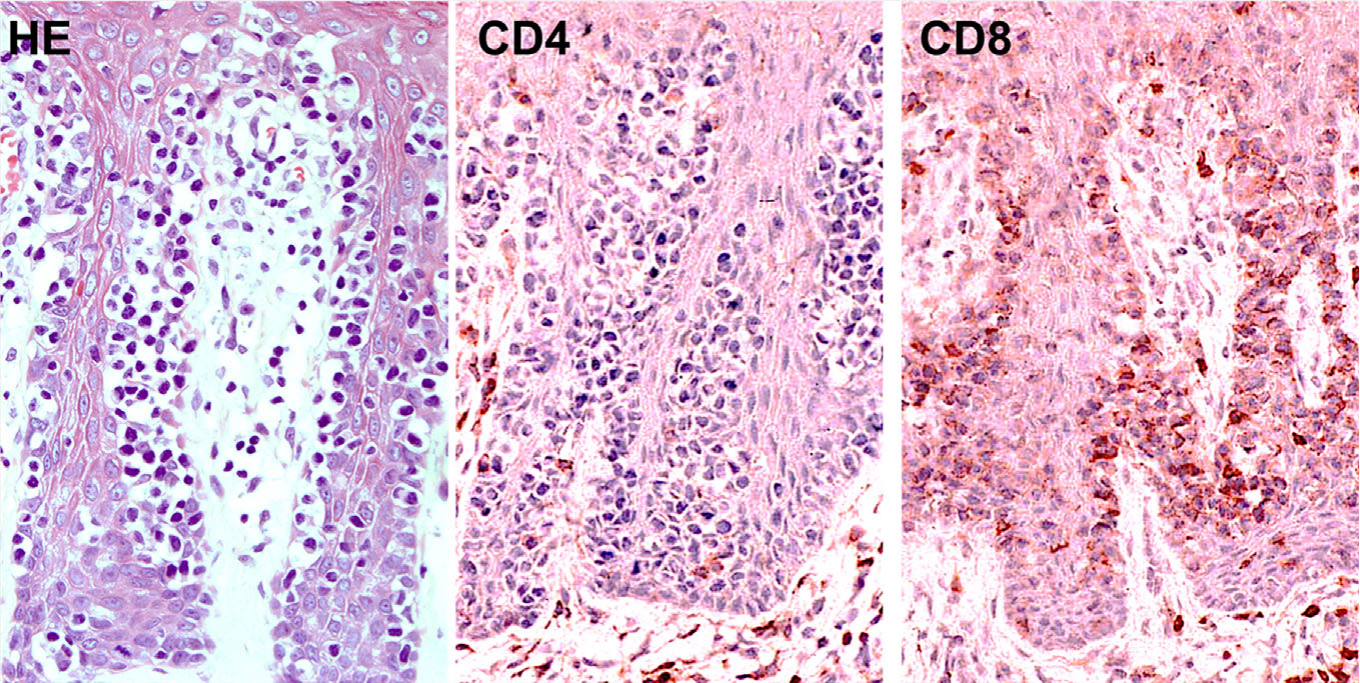
Histopathology (left; hematoxylin and eosin, HE) and immunostaining for CD4 (middle) and CD8 (right) in CD8^+^ pagetoid reticulosis.

It has been reported that some patients with MF have malignant CD8^+^ T cells instead of CD4^+^ T cells. Accordingly, a case of CD8^+^ primary cutaneous peripheral T-cell lymphoma arising from skin T_RM_ cells was also reported (156). Pagetoid reticulosis is histologically characterized by dense infiltration of atypical mononuclear cells in the epidermis that produce a pagetoid appearance. This unique disease is historically divided into the localized type (Woringer-Kolopp disease) and the disseminated type (Ketron-Goodmann disease). However, a case showing progression from the former to the latter was documented (157), and currently, pagetoid reticulosis is regarded as a subtype of MF. In the immunohistochemical phenotype, cases of pagetoid reticulosis can be divided into three subtypes: CD4^+^ (37.5%), CD8^+^ (29.2%), and CD4^-^CD8^-^ (33.3%) types (157). While the single positive types are derived from αβ T cells, the double negative type originates from γδ T cells. It should be noted that one third of pagetoid reticulosis cases are CD8^+^, suggesting that this subtype is an epidermal CD8^+^ T_RM_ cell tumor (**Figure 8**). The pagetoid fashion of this tumor may reflect the nature of skin T_RM_ cells.

## Skin T_RM_ Cells in Fixed Drug Eruption

**Figure 9:**
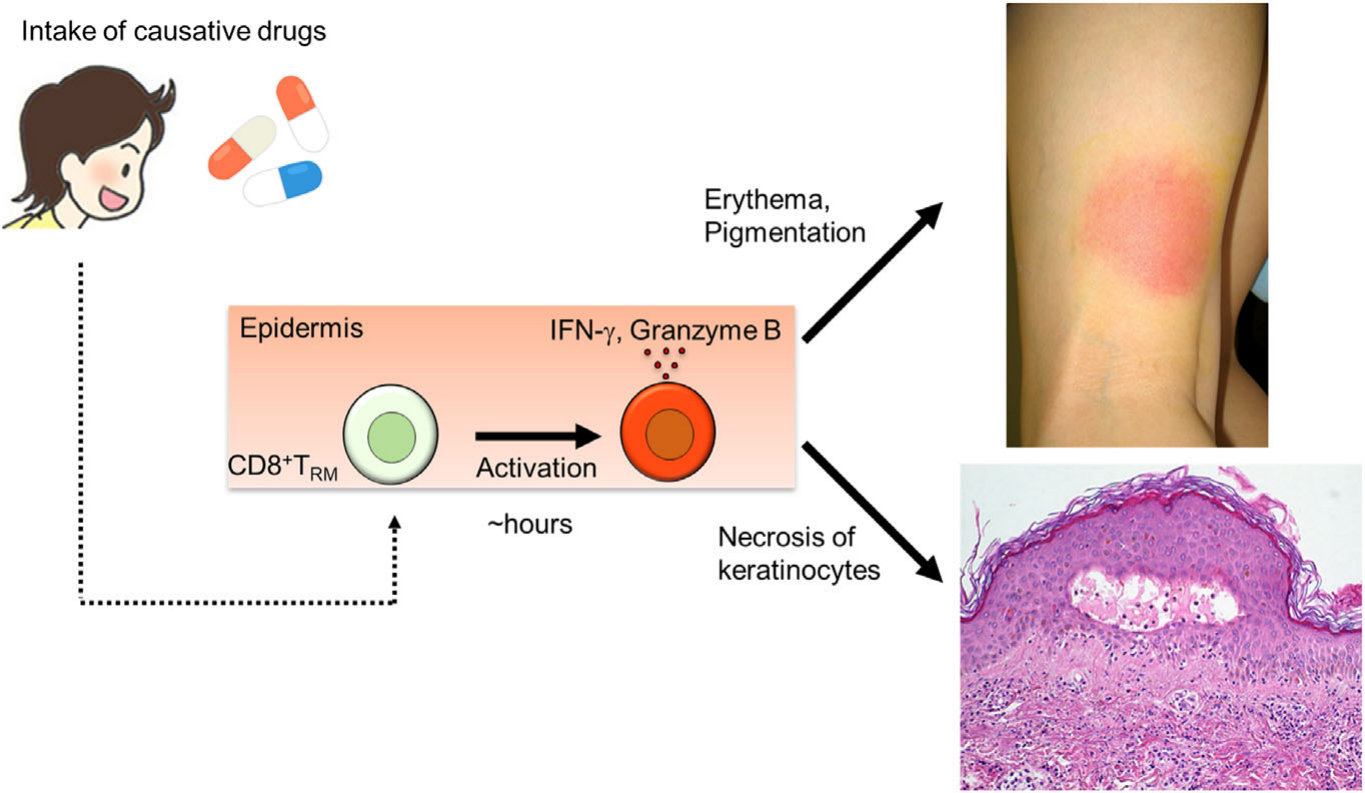
Mechanism of fixed drug eruption.

Fixed drug eruption is induced by skin T_RM_ cells (**Figure 9**). CD8^+^ T_RM_ cells in the epidermis possess an effector-memory phenotype and play a role in development of localized tissue damage in fixed drug eruption (7). These epidermal CD8^+^ T cells constitutively express an early activation marker CD69 even before challenge. A large proportion of these CD8^+^ T cells exhibit immediate effector function as proven by the rapidly increased IFN-γ production after challenge, resulting in localized epidermal injury. In addition, the intracellular cytokine assay *ex vivo* supports the great capability of these T cells to produce IFN-γ (158).

Although reactivation of these CD8^+^ T_RM_ cells is sufficient to initiate the lesion, the recruitment of circulating CD4^+^ and CD8^+^ T cells is necessary to cause extensive tissue damage observed in the fully evolved lesions. The abundance of regulatory T cells in the epidermis of fully evolved lesions would serve to limit aberrant immune reactions. Local IL-15 production from lesional epidermis could maintain the survival of the epidermal CD8^+^ T_RM_ cells even without antigen stimulation over a prolonged period of time (159).

The presence of T_RM_ cells in the epidermis and ocular surface may also play a key role in immune activation and antigen recognition. Some evidence supports the role of T_RM_ cells in Stevens-Johnson syndrome and Toxic epidermal necrolysis, and disease distribution may relate to their site-predominance (160).

## Discussion

One of the important issues on the residency status of skin T_RM_ cells in which what conditions allow T_RM_ cells to emigrate from the tissue is under debate. Skin T_RM_ fate decision seems to be established prior to antigens recognition. Once these naïve T cells encounter with cognate antigen presented by DCs, these pre-conditioned T cells will be ready to become a skin-homing T_RM_ precursor, implying that preconditioned naïve T_RM_ cells are prepared during homeostasis, and skin-homing molecules are imprinted during T cell priming (89). Inflammatory signals from inflamed skin attract these skin-homing cells to the local inflammation site. After entering the skin, local signals induce T_RM_ precursors to differentiate into mature skin T_RM_ cells. The non-differentiated T_RM_ precursors may recirculate between the skin, blood and LNs, where these cells possibly represent circulating memory T cells that have been described as skin recirculating memory T cells in mice (67) or skin-tropic migratory memory T cells in human (14). Interestingly, skin recirculating memory T cells are induced greatly by skin infection but not by intravenous infection (67). Moreover, a very recent study reported that skin T_RM_ could exit their residential skin and rejoin the circulating pool of memory T cells (97). In human *ex vivo* skin experiments, using the nanobody labeling technique also demonstrated that CD8^+^ T_RM_ cells can migrate from the epidermis to the papillary dermis (161). However, whether T_RM_ cells that migrate out of the skin are authentic T_RM_ cells or these cells are skin recirculating memory T cells that intermittently present in skin remains to be elucidated.

Memory T cell populations are more diverse and heterogeneous than initial expectation, and tissue memory responses may be involved beyond the T_RM_ cell population. Recently, a novel concept of tissue memory beyond the role of adaptive immune memory has emerged. The inflammatory memory can be exerted by various cell types and the interaction among these memories across cell lineages and may impact on tissue adaptation and maladaptation (162). It should be noted that the characteristics and behavior of T_RM_ cells are different among barrier tissues, as each barrier tissue has specialized cells residing in each location, as exemplified by keratinocytes in the skin. A chemical allergen like DNFB can persist in the skin for several weeks, especially in keratinocytes around hair follicles, a part of which are slow-cycling epidermal stem cells (99). This remaining allergen in keratinocytes correlate with the number of antigen-specific CD8^+^ T_RM_ cells (99). This epithelial memory may contribute to or instruct immune memory cells, and they coordinate each other to maximize the protection. CD8^+^ T_RM_ cells that we have observed may just only a tip of the iceberg in the process of tissue memory responses.

In several cutaneous diseases, the presence of skin T_RM_ cells has been investigated in the active lesional skin and resolved lesional skin along with non-lesional, normal appearing skin. Unexpectedly, in the active lesion, it is no easy task to identify and enumerate T_RM_ cells, because many T cell populations are intermingled with each other and their activity, residency, and fate cannot be easily expected. For example, the involvement of T_RM_ cells in the recurrent lesions of psoriasis and fixed drug eruption are well known. However, it remains a matter of debate whether the cells with T_RM_ markers in the active lesions belong to T_RM_ cells. We have only limited information on the activity and residency of these cells in relation to the clinical significance.

In our clinical study in psoriasis patients, the cells with T_RM_ markers were increased in the active skin lesion and decreased after the systemic treatment with anti-IL-17A mAb, although they were relatively resistance to the treatment compared to the non-T_RM_ cells (142). In addition, T cells bearing T_RM_ markers in the active lesion were capable of producing pathogenic cytokines, such as IL-17A, and were possibly related to the unfavorable disease course (70). In active skin lesion, CD8^+^CD103^+^ cells tended to be present in the middle to upper epidermis, while they were located at the basal layer in the resolved skin and non-lesional skin of psoriasis. Therefore, T_RM_ cells or T_RM_ marker-bearing cells behave as effector cells and likely serve as crucial effectors in psoriasis pathology. Further investigations on their dynamics, detailed functions, and residency are required. Furthermore, to see the disease specificity of these T_RM_ cells, T_RM_ characterization in atopic dermatitis is in progress in our laboratory.

## Author Contributions

Concepts: YT. Wrote the paper: YT, PP, TH, and TF. Designed the figures: PP, YT, and TH. Reviewed manuscript: TH and KK. All authors commented on the manuscript. All authors contributed to the article and approved the submitted version.

## Conflict of Interest

The authors declare that the research was conducted in the absence of any commercial or financial relationships that could be construed as a potential conflict of interest.

## Abbreviations

ATLL, Adult T-cell leukemia/lymphoma; CCL, Chemokine ligand; CLA, Cutaneous lymphocyte-associated antigen; CTCL, Cutaneous T-cell lymphoma; CTLs, cytotoxic lymphocyte; CXCL, CXC chemokine ligand; CXCR, CXC chemokine receptor; DCs, Dendritic cells; DETCs, Dendritic epidermal T cells; FABPs, Fatty acid binding proteins; FFA, Free fatty acid; HSV, Herpes simplex virus; IFN, Interferon; IL, Interleukin; iNOS, Inducible nitric oxide synthase; KLRG1, Killer cell lectin-like receptor subfamily G member 1; LN, Lymph node; MF, Mycosis fungoides; MPECs, Memory precursor effector cells; PD-1, Programmed cell death protein 1; PDE4, Phosphodiesterase 4; PD-L1, Programmed cell death ligand 1; S1PR1, Sphingosine 1-Phosphate Receptor 1; SLECs, Short-lived effector cells; SLOs, Secondary lymphoid organs; SS, Sézary syndrome; TCM, Central memory T cell; TEM, Effector memory T cell; TMM, Skin-tropic migratory memory T cell; TPM, Peripheral memory T cell; TRM, Resident memory T cell; Th, Helper T cell; Treg, Regulatory T cell; TCR, T-cell receptor; TILs, Tumor-infiltrating lymphocytes; TIP-DCs, TNF-α iNOS producing dendritic cells; TNF, Tumor necrosis factor; VLA, Very late antigen protein.
